# Detection of coronary microembolization by Doppler ultrasound in patients with stable angina pectoris during percutaneous coronary interventions under an adjunctive antithrombotic therapy with abciximab: design and rationale of the High Intensity Transient Signals ReoPro (HITS-RP) study

**DOI:** 10.1186/1476-7120-10-21

**Published:** 2012-05-21

**Authors:** Daniel Kretzschmar, Christian Jung, Sylvia Otto, Stephan Utschig, Michael Hartmann, Thomas Lehmann, Atilla Yilmaz, Tudor C Pörner, Hans R Figulla, Markus Ferrari

**Affiliations:** 1 Department of Internal Medicine I, Division of Cardiology, University Hospital Jena, Erlanger Allee 101, D-07740, Jena, Germany; 2 Pharmacy of University Hospital Jena, Erlanger Allee 101, D-07740, Jena, Germany; 3 Institute of Medical Statistics, Computer Sciences and Documentation, University Hospital Jena, Bachstraße 18, Jena, D-07743, Germany

## Abstract

**Background:**

Embolization of atherosclerotic debris from the rupture of a vulnerable atherosclerotic plaque occurs iatrogenically during percutaneous coronary interventions (PCI) and can induce myocardial necrosis. These microembolizations are detected as high intensity transient signals (HITS) using intracoronary Doppler technology.

**Presentation of the hypothesis:**

In the presented study we will test if abciximab (ReoPro®) infusion reduces high intensity transient signals in patients with stable angina pectoris undergoing PCI in comparison to standard therapy alone.

**Testing the hypothesis:**

The High Intensity Transient Signals ReoPro® (HITS-RP) study will enroll 60 patients. It is a prospective, single center, randomized, double-blinded, controlled trial. The study is designed to compare the efficacy of intravenous abciximab administration for reduction of microembolization during elective PCI. Patients will be randomized in a 1:1 fashion to abciximab or placebo infusion. The primary end point of the HITS-RP-Study is the number of HITS during PCI measured by intracoronary Doppler wire. Secondary endpoints are bleeding complications, elevation of cardiac biomarkers or ECG changes after percutaneous coronary interventions, changes in coronary flow velocity reserve, hs-CRP elevation, any major adverse cardio-vascular event during one month follow-up.

**Implications of the hypothesis:**

The HITS-RP-Study addresses important questions regarding the efficacy of intravenous abciximab administration in reducing microembolization and periprocedural complications in stable angina pectoris patients undergoing PCI.

**Trial registration:**

The trial is registered under http://www.drks-neu.uniklinik-freiburg.de/drks_web/:DRKS00000603.

## Background

Following percutaneous coronary intervention (PCI) an increase of cardiac marker enzymes is relatively often observed and associated with reduced coronary flow velocity reserve (CFVR) [1]. Serum concentration of cardiac troponin I (cTNI) was reported to be increased in 30–40% of cases [2]. The troponin rise is the result of myocardial necrosis during PCI induced by embolization of atherosclerotic and thrombotic debris during balloon or stent inflation [3]. Troponin elevation is associated with dismal prognosis in patients with unstable angina [4] and PCI [5]. Periprocedural coronary microembolization occurs in about 25% of all PCIs. The incidence ranges from 0 to 70%, depending on the method of assessment [6]. Coronary microembolization is a common event during several phases during PCI. Even passing of the stenosis with the stent or balloon may be a vulnerable phase [7].

The consequences of coronary microembolization are microinfarctions with an inflammatory response, contractile dysfunction, perfusion-contraction mismatch, and reduced CFVR [8]. The number of microparticles correlate to the size of myocardium at risk in patients with ST-elevation myocardial infarction [9].

The intracoronary Doppler guidewire is a feasible device for detection and quantification of microembolism occurring during PCI [10]. In a previous study we could demonstrate that the incidence of procedural associated non-ST elevation myocardial infarction (pNSTEMI) is correlated to the frequency of Doppler-detected microemboli [7].

Several clinical studies unravelled that cardiac biomarker elevations directly correlated with the extent of myocardial necrosis [11]. In patients with pNSTEMI the myocardial damage represents up to 5% of the left ventricular mass [12].

The progressive contractile dysfunction results from an inflammatory reaction to microinfarctions. Elevation of high-sensitivity C-reactive protein (hs-CRP) levels providing prognostic information for patients receiving PCI [13] and could be derived directly from inflammation or from secondary reaction to microinfarctions due to microembolization [14]. This inflammation marker could be used as a predictor for early complications after stent deployment [15].

### Presentation of the hypothesis

The mouse monoclonal antibody abciximab against the platelet receptor glycoprotein IIb/IIIa (GPIIb/IIIa) is able to inhibit platelet aggregation by more than 80% [16]. In patients with acute myocardial infarction abciximab was able to improve myocardial microcirculation and reduce infarct size due to dissolution of thrombi and microemboli [17]. Therefore we hypothesize that abciximab is a possible agent to reduce coronary microembolization in patients with stable CAD undergoing elective PCI.

### Testing the hypothesis

The HITS-RP study is a prospective, double-blinded, randomized, placebo controlled trial in patients with coronary artery disease (CAD) undergoing PCI. The study goal is to determine the efficacy of intravenous abciximab bolus application with subsequent 12-hour intravenous infusion in reducing high intensity transient signals (HITS) compared to placebo. The trial is registered under http://www.drks-neu.uniklinik-freiburg.de/drks_web/:DRKS00000603.

### Primary and secondary outcome

The primary study end point of the HITS-RP study is the incidence of HITS during PCI. Secondary endpoints are changes in CFVR, cardiac biomarkers (cTNI, CK, CK-MB) and hs-CRP in comparison to the initial values, ECG changes, bleeding complications due to the additional thrombocyte inhibiton, and any type of major adverse cardiac or vascular event during one month follow-up. Bleeding complications will be assessed according to the GUSTO criteria (a-severe or life-threatening; b-moderate; c-mild) [18].

### Patient population

The prospective study will include 60 consecutive patients with CAD and elective PCI. Recruitment will commence in may 2012. The Institutional Ethics Committee of University Hospital of Jena approved the study protocol.

#### Inclusion criteria

Patients are eligible for the study if they are between 18 and 80 years of age. Inclusion criteria are stable angina pectoris and written informed consent.

#### Exclusion criteria

The exclusion criteria were (1) increase of cardiac markers before PCI, (2) left bundle-branch block, (3) terminal renal insufficiency, (4) hypothyroidism, (5) skeletal muscle injury, (6) chronic occluded target artery, (7) bifurcation lesion, (8) in-stent restenosis, (9) planned multivessel intervention, (10) side branch occlusion or (11) prolonged vasospasm and (12) any contraindication for antiplatelet medication.

### Randomization

**Figure 1:**
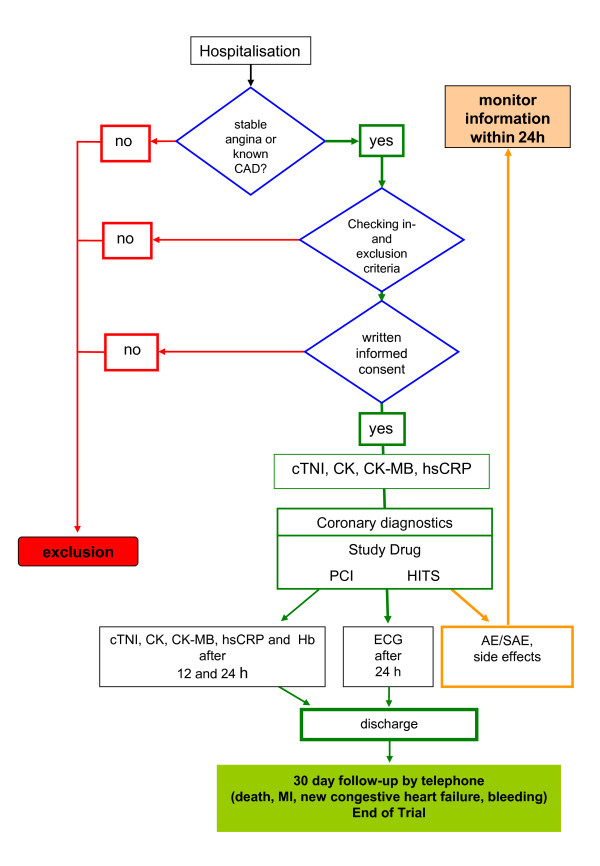
ReoPro HITS flowchart.

Patient randomization is performed centrally with a randomization ratio of 1:1 (n = 30 patients per group). Patients will be randomized by blockwise randomization with a fixed block size of 6 using Datinf randList 1.2. The random number generator is based on the algorithm of Park and Miller with Bays-Durham correction. Allocation concealment is done in the pharmacy. Medication will be delivered in black syringes with a label only containing study name and patients number (Figure 1).

### Power calculation

Based on the results of previous studies [7] we expect 27 HITS on average in the placebo group and 18 HITS in the treatment group. Assuming a standard deviation of 12 in both groups, we will need a sample size of 29 patients to detect the treatment effect with a power of 80% at a two-sided alpha-level of 0.05. To account for drop-outs we have chosen to include 30 patients per group in the study.

### Patient treatment

All patients receive 100 mg acetylsalicylic acid once daily before PCI and thereafter according to the standards of good clinical practice. Those patients not already taking clopidogrel medication will receive 600 mg immediately after PCI and afterwards 75 mg once daily. A heparin bolus of 70 IU Heparin/kg body weight is given following the insertion of the arterial sheath. All other medication is given at the discretion of the attending cardiologist. After diagnostic angiography with knowledge of the coronary anatomy patients are planned to undergo balloon dilatation with implantation of a stent for a single stenosis. The choice of stent (bare-metal or drug-eluting stent) is left to the discretion of the attending cardiologist. Patients will be randomly assigned to the glycoprotein IIb/IIIa receptor antagonist abciximab or placebo. The study drug will be given as an i.v. bolus of 0.25 mg/kg body weight 10 minutes before PCI followed by a continuous infusion at 0.125 μg/kg/min initiated immediately after the bolus and continued for 12 h. A Doppler guidewire is positioned 1 to 2 cm distal to the stenosis for PCI. At this position the average peak velocity (APV) is recorded. Before and after each interventional step CFVR is measured as the ratio of maximum APV and baseline APV. Maximum hyperemia will be induced by intracoronary injection of 30–40 μg adenosine. The number of HITS will be measured as previously described [7].

After PCI, removal of the sheath is performed according to local practice. Care must be taken to ensure adequate hemostasis.

After 12 and 24 h creatine kinase (CK), creatine kinase-MB (CK-MB), cardiac troponin I (cTNI), hemoglobin (Hb), hs-CRP will be determined and after 24 h also an ECG will be recorded. At 30-day follow-up all cause mortality, myocardial infarction, angina pectoris, new congestive heart failure, and bleeding complications will be assessed. This is the end of the trial.

### Data and statistical analysis

The primary end point will be analyzed according to the intention-to-treat principle using the independent-samples t-test. In addition per-protocol analysis and to adjust for risk factors (such as diabetes, hypertension, smoking, cholesterol level, sex) multivariate regression analysis will be performed. The secondary endpoints are compared using Student’s t-test, Mann–Whitney U test, chi-square test and Fisher’s exact test as appropiate and by multivariate regression models.

All data will be collected independently in a blinded database by an external data management facility (2conduct clinical trials). A p-value of <0.05 will be considered as statistically significant. Data analysis will be performed using SAS 9.3 (SAS Institute Inc.).

## Discussion

Coronary microembolization is a frequent event in ischemic heart disease, occurring artificially during coronary interventions. Especially patients with multivessel coronary artery disease, more than 20 microemboli, and high inflation pressure during PCI are at risk for pNSTEMI [7]. Thus, the protocol of the HITS-RP study is focused on patients undergoing elective PCI for stable CAD.

It is important to note that data for the use of filter devices during PCI were disappointing and no significant protection against microembolization was achieved in previous studies [19]. In consequence novel treatment strategies of preventing patients from pNSTEMI are desired.

Numerous studies demonstrated the effect of anti-platelet agents in patients with stable angina pectoris or acute coronary syndrome for attenuation of myocardial damage and reduction of adverse events [20,21]. However, it is still unclear whether the respective agent is able to reduce the formation of coronary microemboli. The glycoprotein IIb/IIIa receptor antagonist tirofiban reversibly suppressed HITS in the cerebrovascular circulation [22]. Thus, this clinical study is designed to identify a target for drug therapy of coronary microembolization and its consequences. In previous clinical trials, the benefits of abciximab infusion were achieved at the risk of increased bleeding [16]. It is necessary to mention that also the safety of abciximab infusion when added to antiplatelet therapy with aspirin and clopidogrel is an important question of the HITS-RP study.

### Limitations

Due to the small study size results will be interpreted with caution and in case of reduction of HITS a larger clinical trial will have to follow. Due to statistical reasons, we do not expect a difference in the composite major adverse cardiac event rate (death, reinfarction, target vessel revascularization, new congestive heart failure) between the two study arms.

### Clinical implications of the hypothesis

Clinical findings suggest that coronary microembolization and pNSTEMI are frequent periprocedural complications. Relevant numbers of microemboli during cardiac interventions could also be detected in the cerebrovascular circulation [23].

Administration of abciximab could protect the myocardium and maybe also the cerebrovascular system. It may reduce the incidence of myocardial damage and also the inflammatory reaction associated with myocyte necrosis. Because of its ease administration and general availability this treatment approach could have a high potential in clinical practice.

## Abbreviations

APV: Average peak velocity; CAD: Coronary artery disease; CFVR: Coronary flow velocity reserve; CK: Creatinine kinase; CK-MB: Creatinine kinase muscle/brain; cTNI: Cardiac troponin I; ECG: Electrocardiogram; GP IIb/IIIa: Glycoprotein IIb/IIIa; Hb: Hemoglobin; HITS: High intensity transient signal; hs-CRP: High-sensitivity C-reactive protein; PCI: Percutaneous coronary intervention; pNSTEMI: Procedural associated non-ST elevation myocardial infarction.

## Competing interests

The authors declare that they have no competing interests.

## Authors’ contributions

DK designed the study protocol and drafted the manuscript. SO and AY designed the study protocol. SU and TCP participated in study design. TL performed statistical analysis. MF conceived of the study and helped to draft the manuscript. HRF and CJ conceived of the study. All authors reviewed the manuscript. HRF and MF are the scientific supervisors of the project. DK is the corresponding author. All authors read and approved the final version of the manuscript.
